# Comparison of HFOV and conventional ventilation in H1N1 influenza ARDS

**DOI:** 10.1186/cc13483

**Published:** 2014-03-17

**Authors:** S Jog, S Sable, D Patel, N Kapadnis, P Rajhans, P Akole, B Pawar, B Bhurke, M Kothari, S Gururaj

**Affiliations:** 1 Deenanath Mangeshkar Hospital and Research Center, Pune

## Introduction

HFOV is a promising rescue therapy for refractory hypoxia in severe ARDS.

## Methods

This is a retrospective comparative study. We retrieved data for all patients with H1N1 influenza-related severe ARDS treated in the ICU during October 2009 to April 2013. Our ICU had only one HFOV machine during the pandemic. Patients were divided into two groups: HFOV group (received HFOV at first eligibility) and conventional lung protective ventilation (CLPV) group (did not receive HFOV at first eligibility due to nonavailability of HFOV). Eligibility criteria for rescue therapy by HFOV were: P/F ratio ≤100; PEEP needed above 12 cm; Pplat ≥30 cm on CLPV. There was no selection or omission bias for HFOV application and HFOV was applied to the first eligible patient. Patient demographic data, laboratory parameters, hemodynamic variables, and oxygenation and ventilator settings were recorded while on CLPV at first HFOV eligibility in all patients.

## Results

The total of 43 patients who met the rescue therapy criteria were further grouped into the HFOV group (24 patients) and the CLPV group (19 patients) depending upon modality of ventilation received after satisfying first-time HFOV eligibility criteria. Both groups were comparable for differences with Fisher's *t *test for qualitative variables and ANOVA for quantitative variables (Table 1), except for higher mortality in the CLpV group (16/19 (84.4%) vs. 12/12 (50%), *P *= 0.026). On logistic regression analysis to find independent variables differentiating the two groups, mortality was higher in the CLPV group (*P *= 0.02, odds ratio (CI) 71.60 (1.85 to 2,766.59)) compared with the HFOV group.

**Table 1:** 

Variable	CLPV *(n = *19)	HFOV (*n *= 24)	*P *value
Age	41.15	32.83	0.07
SOFA	4.95	4.62	0.41
Pplat	29.57	30.46	0.26
O.I.	38.88	35.70	0.54
P/F	70.63	76.79	0.50
PCO_2_	59.89	59.92	0.99
Death	16	12	0.02

## Conclusion

HFOV when applied as rescue therapy for refractory hypoxia due to severe ARDS caused by H1N1 influenza pneumonia is associated with better outcome compared with CLPV.

